# Validity Evidence of an Epidemiological Oropharyngeal Dysphagia Screening Questionnaire for Older Adults

**DOI:** 10.6061/clinics/2020/e1425

**Published:** 2020-01-06

**Authors:** Hipólito Virgilio Magalhães Junior, Leandro de Araújo Pernambuco, Renata Veiga Andersen Cavalcanti, Kenio Costa Lima, Maria Angela Fernandes Ferreira

**Affiliations:** I Departamento de Fonoaudiologia, Centro de Ciencias da Saude, Universidade Federal do Rio Grande do Norte (UFRN), Natal, RN, BR; II Departamento de Fonoaudiologia, Centro de Ciencias da Saude, Universidade Federal da Paraíba (UFPB), Joao Pessoa, PB, BR; III Departamento de Odontologia, Programa de Pos-Graduacao em Saude Coletiva (PPGSCol-UFRN), Universidade Federal do Rio Grande do Norte (UFRN), Natal, RN, BR

**Keywords:** Deglutition Disorders, Aging, Mass Screening, Epidemiology, Validation Studies

## Abstract

**OBJECTIVE:**

This research aimed to identify evidence of validity of a self-reported Oropharyngeal Dysphagia screening questionnaire for older adults based on test content, response processes, internal structure, relations to other variables, and reliability.

**METHOD:**

This is a nonrandomized, cross-sectional study employing the concepts and principles of the Standards for Educational and Psychological Testing. Data were collected from 644 community-dwelling older adults (both genders, age: ≥60 years) who agreed to participate in some steps of the validation process. Statistical methods obtained the content representation of the construct, internal structure validation, discriminant and convergent evidence, and reliability, using a 5% significance level.

**RESULTS:**

The screening tool was re-specified in nine questions that provided the best fit and robust reliability, with proper discriminant and convergent evidence.

**CONCLUSIONS:**

The screening questionnaire presented valid and reliable results to identify oropharyngeal dysphagia symptoms in older adults, highlighting the importance of the validation process based on the standards to construct an epidemiological instrument.

## INTRODUCTION

Oropharyngeal dysphagia (OD) is a swallowing disorder that may indicate disorder in oral, pharyngeal, or oropharyngeal functioning during the swallowing process. It is associated with noticeable symptoms reported by the individual on ingesting food (1), which presents issues surrounding weight loss, malnutrition, dehydration, and episodes of aspiration pneumonias. These issues are of great concern in the healthcare of older adults. OD has an impact on public health as it can increase the morbidity and mortality of older adults (2).

This clinical condition can increase functional disability and frailty, hospital readmissions, mortality, and the institutionalization of older adults. OD has been recognized as a critical geriatric syndrome by two European Societies (2,3).

The prevalence of OD in older people who live in the community varies between 11% and 38% (4-8). However, the studies that have reported prevalence did not use screening instruments for epidemiological diagnosis with validity evidence for the older adult population (9).

For a screening instrument to become both epidemiologically relevant and scientifically robust, it must be developed and psychometrically tested for its validity. It should measure what it intends to achieve and must be reliable in producing consistent and reproducible data (10).

Hence, this study aimed to identify the evidence of validity in constructing an epidemiological self-reported Oropharyngeal Dysphagia screening questionnaire for older adults.

## MATERIAL AND METHODS

This nonrandomized and cross-sectional study evaluated the evidence of validity according to the test content, response processes, internal structure, relations to other variables and reliability (11).

Data were collected from March 2013 to April 2017. The adjusted versions for each stage of this validation process were used. Participants of either gender aged 60-95 years with preserved cognitive functioning were included in the study. The mean age varied depending on when the data was collected.

This tool aimed to be an epidemiological screening instrument for OD in older adults with no specific diseases. This process excluded participants who were previously diagnosed with comprehensive, psychiatric, neurological, and neurodegenerative disorders or head and neck cancer, who were tracheostomized, who had moderate-to-severe hearing loss (with no hearing aids), or who used feeding tubes.

The study participants were recruited by convenience from social centers for older adults, one long-term care institution for older adults, and the waiting room of a Geriatric and Otorhinolaryngology Outpatient in the University Hospital, Natal, Rio Grande do Norte, Brazil.

### Evidence of validity based on test content

An expert technical group (ETG) composed of five researchers (who are the authors of this study) created the initial version of the questionnaire through a free association of ideas based on the most common signs and symptoms of swallowing disorders.

The theoretical basis of this study defined OD as a swallowing disorder resulting from a broad spectrum of disturbances in the interaction between the oral and pharyngeal phases. This disorder can lead to complications in pulmonary and nutritional status (12).

The ETG considered the following qualitative aspects: the educational level of the elderly; how long it took for respondents to answer the questions; the presence of ambiguous words, vague terms, or (medical) jargon; and items with two or more questions.

The initial version of the questionnaire consisted of 17 items focusing on sensations and perceptions related to swallowing disorders of the oral and/or pharyngeal phases. This was judged by a multidisciplinary expert committee (EC) who evaluated the adequacy of the questions. The EC consisted of 22 speech pathologists (3 gerontologists, and 19 dysphagia specialists), five geriatricians, two dietitians, one nurse, one otorhinolaryngologist, and one gastroenterologist.

The analysis used the Content Validity Index by Item (CVI-I) and the Content Validity Index (CVI) to verify the level of agreement of the EC evaluations, estimated as ≥0.80 and ≥0.90, respectively. The conceptual framework refinement in a single round of consultations was considered (10). The ETG also considered the suggestions of the EC to modify or insert some questions (13,14).

### Evidence of validity based on response processes

After the analysis by the EC, a second version with 16 items was applied to 40 older adults from different social strata (Table 1), according to criteria from a previous study (10).

The participants were asked about the clarity of each item, followed by a request to repeat how they had understood it in a loud voice. Indirect strategies were used to observe their response time and non-verbal reactions to identify problematic sentences that had issues surrounding their meaning or interpretation (15).

### Evidence Based on Internal Structure

This version of the epidemiological screening questionnaire, with 14 questions, was applied to 211 older adults, of whom 63 (29.9%) were male and 148 (70.1%) were female (mean age=71.2; SD=7.8).

This evidence was obtained by confirmatory factorial analysis by structural equation modeling (CFA-SEM). The grouping of the questions was organized into two factors, based on highlights from the literature about what each item could mean as an outcome of deglutition disorders (Table 2). This resulted in a hypothetical model (16).

**Figure 1:**
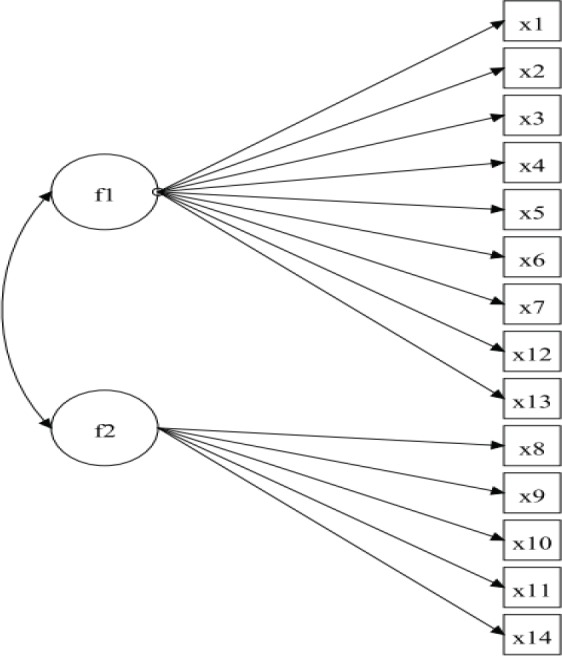
The hypothetical, theoretical framework screening model. This unidimensional measurement model was defined with 14 observable variables: x1: Oral regurgitation; x2: Multiple swallows; x3: Swallowing with effort; x4: Longer time to eat; x5: Limit food intake; x6: liquids with meals; x7: Nasal regurgitation; x8: Throat clearing; x9: Wet voice; x10: Choking; x11: Recurrent pneumonia; x12: Weight lost; x13: Painful swallowing; and x14: Fatigability. These variables are grouped into two factors (latent variables): f1: non-efficiency swallowing; f2: unsafe swallowing.

Thus, a unidimensional measurement model was defined (Figure 1) with 14 observable variables, grouped into the following two factors (latent variables): non-efficiency swallowing (f1) and unsafe swallowing (f2).

The CFA-SEM was performed using MPlus 8.0 version software, applying the Robust Weighted Least Squares (WLSMV). This performs well with small samples (n≥200) and ordinal categorical variables (23).

Verification of the polychoric correlation matrix was initially performed. The fit indexes to evaluate the initial model included lowest and non-significant chi-square (χ^2^), normed chi-square (χ^2^/df) ≤2, Root Mean Squared Error of Approximation (RMSEA) ≤0.06, Weighted Root Mean Square Residual (WRMR) ≤1.0 (24), Comparative Fit Index (CFI) ≥0.95, and Tucker-Lewis Index (TLI) ≥0.96 (25).

The quality of each factor of the model was also analyzed to evaluate the possibility to eliminate any of the variables, considering: an average variance extracted (AVE) ≥0.60, a mean of the standard factorial loads (Ẍλ) ≥0.70, and composite reliability coefficients (CR) ≥0.91 for a 10-variables-model (or close to 0.90 if the adjusted model becomes less than 10 variables) (26).

### Evidence Based on Relations to Other Variables and Reliability

This stage considered convergent and discriminant validity. A well-fitting model with nine items was applied to 393 older people (mean age=70.4; SD=7.9), with 175 (44.5%) males, and 218 (55.5%) females.

When considering convergent validity, the Associated Factors Questionnaire was used with dichotomized responses (&quot;yes^1^ or no^0^&quot;). This questionnaire addressed the presence of comorbidities, discomfort symptoms, negative lifestyle, and inferior self-perception of functionality. This was measured by the sum of the total scores of the worst outcomes.

The questionnaire scores ranged from 0 to 11 and were scored according to: self-reported clinical history of stroke (4,27), heart disease (4), thyroid problems (4), diabetes (27), depression (4,6,27); voice problems, neck and throat tension, shoulder tension; self-reported inactivity (4), lack of exercise (4), and eating alone (28).

Convergent validity was verified based on the assumed hypothesis that older people with more referred associated factors had worse screening results, as analyzed by the Spearman’s coefficient of correlation *(*ρ).

Discriminant validity was assessed by the validated and adjusted Rosenberg Self-esteem Scale (RSES) for Brazilian older adults (29). This was used with part of the sample (n=110; mean age=71.8; SD=8.2), including 30 (27.3%) males and 80 (72.7%) females. The objective was to prove that there was a weak or non-correlation through the Spearman’s coefficient.

The reliability of the test-retest reproducibility was assessed using part of the sample (n=75; mean age=70.5; SD=7.1), in which 28 (37.3%) were males, and 47 (62.7%) were females. They came back to a second interview within a period of 5 to 14 days (25).

The statistical reliability analysis used the intraclass correlation coefficient (ICC) of the instrument (scored from 0 to 18) (30), the Standard Error of Measurement (SEM) and the Smallest Real Difference (SRD) (31). The internal consistency was tested using Cronbach’s alpha ≥0.70 (25).

### Ethics

The present study complies with all recommended ethical guidelines for research involving human subjects and was approved by the Institution’s Ethics and Research Committee (approval number: 1,144,297).

## RESULTS

### Evidence of validity based on content

The CVI was 0.72, below the considerable minimum value to affirm that the instrument has good conceptual representation, pertinence, clarity, and comprehensiveness of the questions. This allowed the verification of the CVI-I of each question (Table 3) followed by descriptions of the adjustments made.

The ETG eliminated four questions from the first version (2, 10, 12, and 15) for presenting low CVI-I (first column of Table 3), without providing suggestions for modifications that may improve clarity. The EC suggested restructuring other questions for being considered theoretically relevant.

Despite presenting a good CVI-I, the experts eliminated the fourth question (first column of Table 3) due to focusing on chewing. According to the authors’ understanding, chewing is a subject that involves other domains beyond screening for OD and is the study of another screening instrument (32).

### Evidence of validity based on response processes

Observing the new order of the 16 items (fourth column of Table 3), the ETG deleted two questions. The ninth question was deleted because more than 70% of the older adults misunderstood if the question was asking about poor consumption of liquid or their weak preference to drink water. The 16^th^ question was deleted for generating confusion with the 15^th^ question.

The questions from the second version (fourth and fifth column of Table 3) that generated the most doubt or confusion or created delay in responding among all groups were 01, 02, 05, 08, 10, 12 and 13. Due to the considerations and reflections regarding the clarity and construction of the questions, the authors decided to modify and reorder the questions based on a sequence of symptoms to facilitate their interpretation with small adjustments in the syntactic construction (Table 2).

When completing the questionnaire, the ETG decided to establish three response options: “No^0^,” “sometimes^1^,” and “always^2^.” However, the question related to weight loss required only two options: “No^0^” and “Yes^1^.” This resulted in the third 14-question-version screening tool.

### Evidence of validity based on the internal structure

The 14-question-model with two factors had no proper adjustment for factor 1 (AVE=0.45, CR=0.86; Ẍλf1=0.58), which reflected small loads of some items, resulting in the elimination of variables x1, x4, x5, x6, and x7.

**Figure 2:**
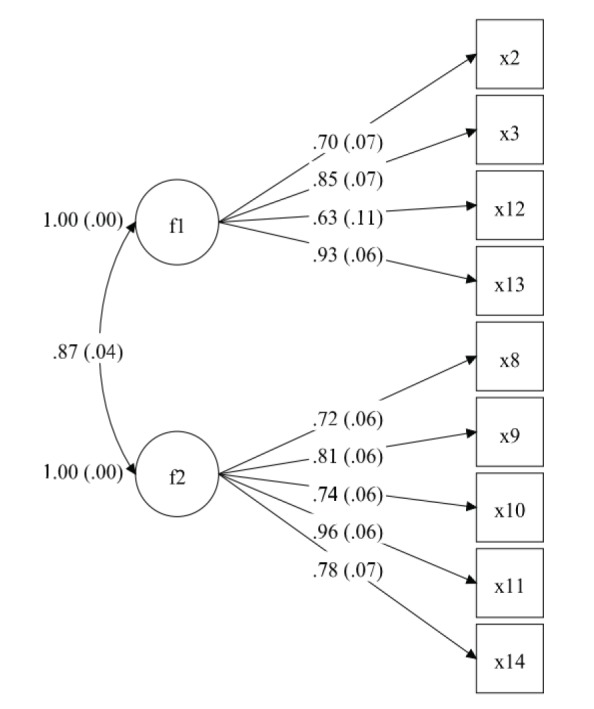
The second-order model of measurement of the constructs according to CFA. The fit model was adjusted with 9 observable variables: x2: Multiple swallows; x3: Swallowing with effort; x8: Throat clearing; x9: Wet voice; x10: Choking; x11: Recurrent pneumonia; x12: Weight lost; x13: Painful swallowing; and x14: Fatigability. These variables are confirmed into two factors (latent variables): f1: non-efficiency swallowing; f2: unsafe swallowing. Correlation between factors f1 and f2 was classified as strong (Φ=0.87; SE=0.04).

After the adjustments, a new structural model reached the well-fit model when compared to the first model (Table 4), as shown in Figure 2.

There were better index precision measures in this adjusted model with less than 10 variables (AVEf1=0.62; CRf1=0.86; Ẍλf1=0.85; AVEf2=0.65, CRf2=0.90; Ẍλf2=0.80; *p*<0.001), indicating good construct reliability and adequate convergent validity.

The 9-item version of the instrument showed robust results for each item as observed from test-retest reliability (ICC=0.83, CI 0.74-0.89, *p*<0.001, SEM=1.17, SRD=3.25) and high internal consistency (α=0.90).

### Evidence Based on Relations to Other Variables

The convergent validity had a positive and moderate correlation (*ρ*=0.43; *p*<0.001) but the discriminant evidence did not demonstrate correlation (ρ=-0.06; *p*=0.6).

## DISCUSSION

The results presented in this study are the first to investigate the evidence of validity for the OD screening questionnaire. The investigation was based on the recommended guidelines by the standards that suggest robust premises to be followed from a psychometric perspective (11).

This self-reported OD screening questionnaire is designed for older adults living in the community with asymptomatic or initial symptoms. The questionnaire was intended to identify the initial diagnosis of this clinical condition, requiring an immediate diagnostic confirmation, leading to a therapeutic decision (33).

Acquiring evidence of validity, based on content, was an essential step towards obtaining accurate results. The process included the quantitative and qualitative evaluation of each question, along with suggestions from the participants for modifications and further inclusion of relevant items to develop an epidemiological screening tool that could be applied in the older adult population.

The results analysis allowed for the adjustment of semantic, syntactic, and contextual aspects of the initial versions of the instrument. This was essential for the clarification and representativeness of the instrument’s relevance to screen for OD.

The quantitative and qualitative evaluations from the ETG, the data from the EC, as well as information from the sample in the response processes favored the modification of the questions. The changes resulted from integrating the given suggestions with empirical observations discussed according to the construct definition (13).

Regarding the evidence of validity related to the response processes, not one screening instrument for OD in this population described this step; therefore, comparisons of the results to other studies was not possible (9). Two studies (34,35) demonstrated their screening tool validation. The first (34) describes a preliminary tool that requires a confirmatory factor analysis to finish its construct validation based on its internal structure. The second (35) provided no evidence on the response processes that collected data on the reactions of the respondents when presented with the questions or the internal structure.

The results emphasize the importance of including detailed descriptions of these steps in studies that are aimed at obtaining evidence of validity from diagnostic tools. This should be considered from the external perception of experts (e.g., the EC and ETG) and the internal perception of who is under evaluation (36).

The findings were relevant, highlighting the importance of describing how the screening tool was constructed and adjusted. This favored the perception of continuous movement in the process of obtaining evidence of validity with interpretations that generated valid and reliable arguments (15).

Evidence of validity, related to the internal structure, well-fitted in nine questions, presented satisfactory quality of the adjustments that were based on the recommendations of the absolute fit indexes (χ^2^/df; RMSEA) and incremental indexes (CFI; TLI). This was confirmed by robust indexes of internal structure, consistency, and reliability (26,31).

There are self-reported questionnaires in the literature that presented no clear evidence of their validation process for OD screening in older adults. One of these questionnaires is being used as a screening tool. However, the author has described that its use was to document the initial severity of dysphagia and to monitor the response to treatment in people with a wide range of swallowing disorders (21).

Another study (37) considered the population of older adults in the community; however, there were failures in the methodological description within the analysis parameters that were already discussed in the literature to obtain evidence of validity, reliability, and accuracy (11).

Therefore, this OD screening questionnaire may be considered as the first and, for the time being, the only self-reported questionnaire from the epidemiological perspective that can generate information to provide valid results to identify OD in older adult residents of the community who are asymptomatic or presenting initial symptoms.

The tool’s reliability measures were consistent. This indicated that there was a small variability in the scores between the subjects, thus, resulting in adequate stability, and non-significant measurement error. Hence, there is a good homogeneity of the items concerning the construct (25).

The convergent validity was significant. This confirms what was found in other studies: only a few factors were associated with OD (e.g., depression (5,6,8); stroke; neck, throat and shoulder tension; limited food intake; social withdrawal; perception as inactive; and heart disease with complaints of difficulty in swallowing (4)).

There are no instruments that present valid and reliable results to establish the prevalence of OD in the older adult residents in the community. The results presented in this study reinforce the need to establish these parameters in population studies.

Regarding the discriminant validity, this screening tool does not have an association with the RSES. This highlights that its measure is empirically unique and represents a phenomenon of distinct interest from this other construct.

A limitation of the present study was the exclusion of older people with cognitive impairment, hearing loss, a history of head and neck cancer, and an absence of satisfactory verbal communication. This can compromise the analysis of the dysphagia scenario in the older adult population living in the community. As the population of older adults with dementia is increasing (37), it is necessary to consider caregivers who can contribute to this screening tool and identify OD in those who lack verbal communication or have cognitive decline.

The final version of this epidemiological screening questionnaire was named according to its initials in the Brazilian Portuguese title: “RaDI” (Rastreamento de Disfagia Orofaríngea em idosos - Self-reported Oropharyngeal Dysphagia Screening for Older Adults) (Appendix).

## CONCLUSIONS

The 9-question RaDI, developed in Brazilian Portuguese, presents evidence of validity based on the test content, response processes, internal structure, and relations to other variables.

This screening questionnaire is a simple and time-effective instrument created for epidemiological purposes to detect OD symptoms and initiate actions aimed at caring for older adults. The instrument can be used by any health professional working with older adults.

## APPENDIX

## SCREENING FOR OROPHARYNGEAL DYSPHAGIA IN OLDER ADULTS - FINAL VERSION* (Rastreamento de Disfagia Orofaringea em Idosos – RaDI).

### Guidelines to the interviewer about the application of the RaDI

Questions should follow the numbered order of the questionnaire. Please, when asking each question, expect the participant to answer “no” or “yes.” Only after says “yes” should you ask if the specific symptom occurs sometimes or always. It is noteworthy that the screening or screening process of this questionnaire is for epidemiological purposes; it requires diagnostic confirmation by referring the elderly respondent to a referral service.

**Table:** 

Questions	No^0^	Yes	
		Sometimes^1^	Always^2^
1. Precisa engolir muitas vezes o alimento para fazê-lo descer? (Do you need to swallow food many times to make it go down?)			
2. Faz esforço para engolir? (Do you have to make an effort to swallow?)			
3. Sente dor ao engolir? (Do you feel pain when swallowing?)			
4. Perdeu peso por ter dificuldade de engolir? (Have you lost weight due to having trouble eating?)	( ) No^0^	( ) Yes^2^	
5. Tem pigarro depois de engolir? (Do you clear your throat after swallowing?)			
6. Sua voz modifica depois de engolir? (Does your voice change after you swallow?)			
7. Tem engasgo depois de engolir? (Do you choke after swallowing?)			
8. Teve pneumonia depois de algum engasgo? ? (Have you had pneumonia after a choking episode?)			
9. Sente cansaço depois de comer? (Do you feel fatigue after eating?)			
Total score			

* The translation of RaDI from Portuguese to English was done for publication purposes without the steps necessary for transcultural translation and adaptation to the English language.

## AUTHOR CONTRIBUTIONS

All authors affirm that they have contributed to the development of the manuscript, were involved in all stages of the development, and have approved the submitted manuscript. Magalhães Junior HV conceived and designed the study, collected the data, performed the analysis, verified the analytical methods, evaluated the study findings, discussed the results and conclusions and wrote the manuscript. Pernambuco LA contributed to the study conceptualization, formal analysis, and validation of the methodology. Cavalcanti RVA supported the study conceptualization, investigation, and methodology and contributed to the visualization and manuscript review. Lima KC contributed to the study conceptualization, formal analysis, methodology, and project administration. He lead the study validation and supervised and provided support in the writing of the original draft, review, and editing. Ferreira MAF contributed to the study conceptualization, formal analysis, methodology, and project administration. She also lead the study validation and supervised and provided support in the writing of the original draft, review, and editing.

## Figures and Tables

**Table 1:** Distribution Criteria of four social groups that were used to evaluate the tool based on the evidence of validity, according to response processes.

Criteria	Group A	Group B			Group C			Group D
Education	HEC	HSC	HSI	HEC	PSI to HSI	HSC	HEI	NFE to HSC
Private health care system	Y or N	Y	Y or N	Y or N	Y	Y	Y or N	N
Income according to neighborhood of residence*	T3	T3 or T2	T3 or T2	T2 or T1	T3 or T2 or T1	T1	T1	T3 or T2 or T1
Male	4	4	4	4				
Female	6	6	6	6				
Age								
Mean (standard deviation) minimum-maximum	69.9 (±6.03) 62-81	66.3(±4.79) 60-78			72.6(±6.04) 64-83			74.4(±4.97) 66-82

Legend: HEC, higher education complete; HSC, high school complete; HSI, high school incomplete; PSI, primary school incomplete; HEI, higher education incomplete; NFE no formal education; Y, yes; N, no; T3, upper tercile; T2, medium tercile; T1, lower tercile.

* This criterion was categorized using nominal mean monthly earnings (in “reais,” the currency of Brazil) of individuals aged ≥60 years who resided in the neighborhoods of Natal and Parnamirim (Rio Grande do Norte, Brazil) according to information from the 2010 Census, which was obtained from the “Instituto Brasileiro de Geografia e Estatística”(IBGE) site. The absolute earnings values were categorized into terciles and formed three strata (T1, T2, and T3).

**Table 2:** Categorization of items according to outcomes of deglutition disorders based on the literature.

Variables	Questionnaire correspondent items	Outcomes
x1	Sente o alimento sair da boca quando engole? (Do you feel food coming out of your mouth when you swallow?)	Oral regurgitation, oral function reduced (4,7,17,18)
x2	Precisa engolir muitas vezes o alimento para fazê-lo descer? (Do you need to swallow food many times to make it go down?)	Multiple swallows (18-20)
x3	Faz esforço para engolir? (Do you have to make an effort to swallow?)	Swallowing with effort (4,21)
x4	Durante as refeições, demora mais tempo para comer? (Do you take longer to eat during meals?)	Longer time to eat or Longer meal time (4,7,17,18)
x5	Deixa de comer algum alimento que acha difícil de engolir? (Do you avoid eating something you find hard to swallow?)	Limit food intake (4,18,21,22)
x6	Precisa tomar líquidos para engolir melhor? (Do you have to drink fluids to swallow better?)	Liquids with meals (18,22)
x7	Percebe coriza (nariz escorrer) depois de comer? (Do you notice that your nose is running after eating?”)	Nasal regurgitation (4,18,19)
x8	Tem pigarro depois de engolir? (Do you clear your throat after swallowing?)	Throat clearing (4,20)
x9	Sua voz modifica depois de engolir? (Does your voice change after you swallow?)	Wet voice (4,17,20)
x10	Tem engasgo depois de engolir? (Do you choke after swallowing?)	Choking (4,7,17,19-22)
x11	Teve pneumonia depois de algum engasgo? (Have you had pneumonia after a choking episode?)	Recurrent pneumonia (7,20)
x12	Perdeu peso por ter dificuldade de engolir? (Have you lost weight due to having trouble eating?)	Weight lost (7,21)
x13	Sente dor ao engolir? (Do you feel pain when swallowing?)	Painful swallowing (4,21)
x14	Sente cansaço depois de comer? (Do you feel fatigue after eating?)	Fatigability (20)

**Table 3:** Validity evidence based on the test content.

Development of the instrument			Evaluation of the instrument by CE	
Order	First version	CVI-I	New order	Second version
1^st^	Do you feel saliva, medicine, liquid, or any other type of food stuck in your mouth or throat?	0.82	3^rd^	Do you feel the need to swallow saliva, liquid, or food many times?
2^nd^	Do you feel the need to remove the food sitting in your mouth or throat?	0.79	-	Eliminated
3^rd^	Do you feel saliva, medicine, liquid, or any other food slip out of your mouth when you swallow?	0,70	1^st^	Do you feel saliva, liquid, or any other food slip out of your mouth during or after swallowing?
4^th^	Do you feel the need to chew a lot to swallow better?	0.85	-	Eliminated
5^th^	Do you have difficulty in moving food with your tongue to swallow?	0.76	7^th^	Do you have to make an effort to swallow?
6^th^	Do you sneeze or feel food going back through your nose when swallowing?	0.88	2^nd^	Do you feel any food or liquid go up to your nose after swallowing?
7^th^	Do you choke or cough when swallowing?	0.82	15^th^	Do you choke after swallowing?
			16^th^	Do you cough when you swallow?
8^th^	Do you feel the need to clear your throat when you swallow?	0.67	4^th^	Do you clear your throat after swallowing?
9^th^	Do you feel it is hard to use your voice when swallowing?	0.46	5^th^	Does your voice change after you swallow?
10^th^	Do you feel secretions in your mouth or throat when swallowing?	0.55	-	Eliminated
11^th^	Do you feel pain/tightness in your throat or chest when swallowing?	0.88	6^th^	Do you feel pain when swallowing?
12^th^	Do you feel your neck is stiff when swallowing?	0.40	-	Eliminated
13^th^	Do you feel tiredness in the mouth, tongue, or throat when swallowing?	0.67	8^th^	Do you feel fatigue after eating?
14^th^	Do you feel mouth dry?	0.91	9^th^	Do you have a feeling of dry mouth?
15^th^	Do you feel shortness of breath when swallowing?	0.49	-	Eliminated
16^th^	Do you take longer to swallow these days?	0.67	10^th^	Has it been taking you longer to eat?
17^th^	Do you feel the need to change or avoid any food to swallow better?	0.85	11^th^	Do you avoid any food that you find hard to swallow?
			12^th^	Do you need to drink liquids to swallow better?*
			13^th^	Have you lost weight due to having trouble eating?*
			14^th^	Have you had pneumonia after a choking episode?*

* More than 50% of the experts suggested including some critical questions for an epidemiological instrument.

**Table 4:** Comparison of CFA-SEM between the hypothetical model and the well-fit model.

Fit measures	14-questions-model	9-questions-model
Chi-square (χ2)	189.83	45.81
Degrees of freedom (df)	76	26
*p*-value	<0.001	0.01
Normed chi-square (χ2/df)	2.5	1.76
RMSEA	0.08	0.06
CI 90%	0.07-1.0	0.03-0.09
CFI	0.86	0.97
TLI	0.84	0.96
WRMR	1.22	0.72
